# Toward elimination of unwanted catches using a 100 mm T90 extension and codend in demersal mixed fisheries

**DOI:** 10.1371/journal.pone.0235368

**Published:** 2020-07-08

**Authors:** Marianne Robert, Fabien Morandeau, Marion Scavinner, Marion Fiche, Pascal Larnaud

**Affiliations:** 1 Ifremer, Unité de Sciences et Technologies Halieutiques, Laboratoire de Technologie et Biologie Halieutique, Lorient, France; 2 Ifremer, Unité de Sciences et Technologies halieutiques, Plouzane, France; 3 Les Pêcheurs de Bretagne, Quimper, France; Department of Agriculture, Water and the Environment, AUSTRALIA

## Abstract

Most European fishing fleets will need to drastically reduce their unwanted catches to comply with new rules of the common fisheries policy. A more practical way to avoid increasing on-board sorting time and issues linked to storage capacity is to prevent unwanted catches in the first place. We assessed the selectivity properties of an experimental fishing gear that combined a 100 mm T90 cylinder with 130 meshes in the extension and a 100 mm T90 codend of 33 meshes (experimental gear) compared to a 100 mm diamond mesh extension and codend (control gear) during commercial trips using twin trawls. Analysis of the relative size composition of catches indicated a significantly higher escapement of small fish of several target species (e.g. *Lepidorhombus whiffiagonis*, *Melanogrammus aeglefinus*, *Raja* spp, and *Lophius* spp) and non-target species (e.g. *Capros aper* and *Gurnards* spp) from the T90 experimental trawl compared to the control trawl (n = 49 hauls), resulting in a significant reduction of unwanted catches of Gadidae, Triglidae, and Caproidae. In contrast, non-negligible commercial losses of small grade target gadoid species were observed. Mixed general linear models showed that the proportion of ray, haddock and anglerfish retained per length class decreased with increased tow duration. The T90 experimental gear will perform at a commercial level when targeting monkfish, megrim, rays and large haddock, however fishers are not likely to use this gear when targeting smaller-bodied species such as cephalopods, small haddock, whiting (*Merlangius merlangus*) and hake (*Merluccius merluccius*), because the gear is likely to allow large numbers to escape. Selectivity studies often focus on a short list of target species; however, catches of non-target species under quota can be problematic for some fisheries. For example, under the implementation of the Landing Obligation catches of boarfish could choke the French whitefish demersal fisheries in the Celtic sea, as France has no national quota for that species. The device tested constitutes an efficient solution to mitigate catches for such non-target schooling fish.

## Introduction

The European Union common fisheries policy, which regulates fishing activities in European waters, was reformed in 2013, resulting in a new regulation, including article 15 on the landing obligation [1]. It stipulates that all catch from the stocks under total allowable catch (TAC) or minimum conservation reference size (MCRS) regulations must be landed, prohibiting the common practice of throwing unwanted catches back into the sea. These discarding practices represent a substantial protein waste and may lead to unsustainable management of marine resources [2].

Spatio-temporal changes in fishing practices and improvement in selecting gear are the two main practices that fishers can implement to comply with the regulation. Numerous studies have focused on testing gear selectivity in recent decades (see reviews of [3, 4]) by using netting with meshes of different sizes and shapes (e.g. diamond, square, T90) in a variety of places on the trawl (e.g. baiting, extension, codend) and/or by adding devices such as escape grids, dispersive floats, separator panels, lights and pingers (see review [5]). The combinations of gear modifications are almost infinite and warrant sea trials to assess their efficiency. Variability in fish behavior among areas, seasons [6, 7] and in the net depending on gear configurations such as vertical openings [8, 9], prevent straightforward extrapolation of results to other case studies.

There are many options that increase size selectivity without increasing mesh size, and one of the easiest to implement is to rotate diamond mesh by 90° (i.e. "T90 mesh"). For a given nominal mesh size, T90 mesh remains more open than diamond mesh during towing [10]. First introduced in the 1990s, T90 mesh has regained interest in recent years. Trials using T90 mesh have been undertaken in various locations, including several European waters, the Mediterranean Sea [11, 12], and in the United States [13], and on a diversity of fleets, from trawlers targeting fish [12, 14], shrimp [11] or Nephrops norvegicus [15] to beam trawlers targeting flatfish [16]. Most experiments tested T90 mesh in the codend, except for Kopp el al. [14], who mounted a 100 mm cylinder of T90 mesh on a subsection of the extension section. Compared to diamond mesh, T90 mesh increases the size selectivity of roundfish species, shrimp and Norway lobster, but tends to decrease the selectivity of flatfish [16]. T90 mesh in the extension piece are mandatory as part of the multiannual management plan for stocks of cod, herring and sprat in the Baltic Sea to reduce the amounts of unwanted catches of cod [17].

In the Celtic Sea, the main target species landed by the French trawling fleet are monkfish (*Lophius piscatorius* and *L*. *budegassa*), haddock (*Melanogrammus aeglefinus*), rays, whiting (*Merlangius merlangus*), megrim (*Lepidorhombus whiffiagonis*), and hake (*Merluccius merluccius*) [18], all of them falling under TAC regulations. In this mixed-fishery context, target species could have incompatible MCRS, which make the optimization of a selective device quite challenging. In 2016, the unwanted catch rate of this fleet was estimated at 30–35%, which equals 19 565–22 951 t of fish (alive or dead) [18]. The main unwanted species discarded in decreasing order are: haddock (both < and > MCRS), lesser-spotted dogfish (*Scyliorhinus canicula*), boarfish (*Capros aper*), gurnard (*Aspitrigla cuculus*, *Chelidonichthys lucerna* and *Eutrigla gurnardus*), rays, and whiting below the MCRS. These species fall under TAC regulations, except gurnard and dogfish.

In this study, we assessed the ability of an experimental fishing gear that combined a 100 mm T90 cylinder of 130 meshes in the extension and a 100 mm T90 codend of 33 meshes to reduce unwanted catch, for both target species below the MCRS and all sizes of fish of non-target species. Comparison of catches between the experimental and control gear were carried out for 6 species using data collected in commercial conditions over an entire year using a twin trawl (n = 49). We tested the effect of covariates on the proportion retained and discuss the results in terms of profitability for the fisheries.

## Materials and methods

**Fig 1:**
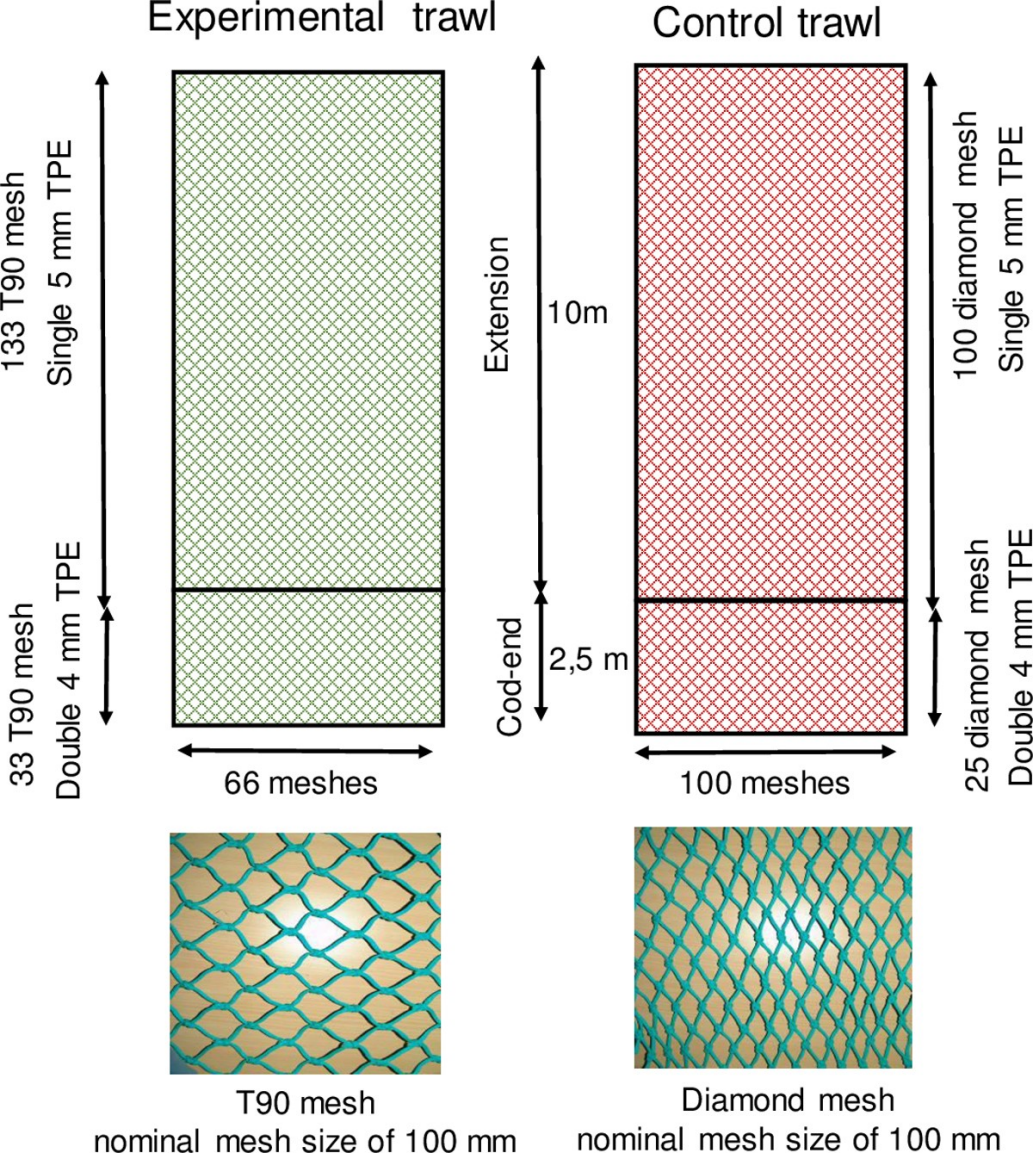
Diagram of the experimental trawl (with the selective device) and the control trawl. TPE means twine polyethylene.

Experiments at sea were conducted on the commercial vessel *An Triskell* (23,95m – 453 KW, 102.27 tx), which was rigged with two warps twin otter trawls, a 36 m ground rope and a 28 m headline. The control trawl corresponded to the gear usually used by the vessel. Its codend was 25 meshes deep, 100 free diamond meshes in circumference and made of 4 mm double twine polyethylene (TPE), with a nominal stretched-mesh size of 100 mm. Its extension was 100 diamond meshes deep and made of 5 mm single TPE, with a nominal stretched-mesh size of 100 mm. The experimental trawl was equipped with T90 netting with a nominal stretched-mesh size of 100 mm, a cylinder of 130 meshes mounted in the extension (single 5 mm TPE) and 33 meshes in the codend (double 4 mm TPE) with a 66-mesh circumference (Fig 1). The rate of assembly between standard diamond mesh and T90 mesh equals three diamond meshes to two T90 meshes. The extensions and codends of both trawls had the same lengths (ca. 10 and 2.5 m, respectively) since 13 meshes in T90 equal 10 diamond meshes in length. Trawl geometry was monitored using the on-board trawl monitoring systems of the vessel to ensure proper functioning of the gear. We estimated the vertical opening height at 2.5–3 m and horizontal wingspread between the doors at 79m. However, as the data were not stored, effect of variation in trawl geometry on the results is unknown.

**Fig 2:**
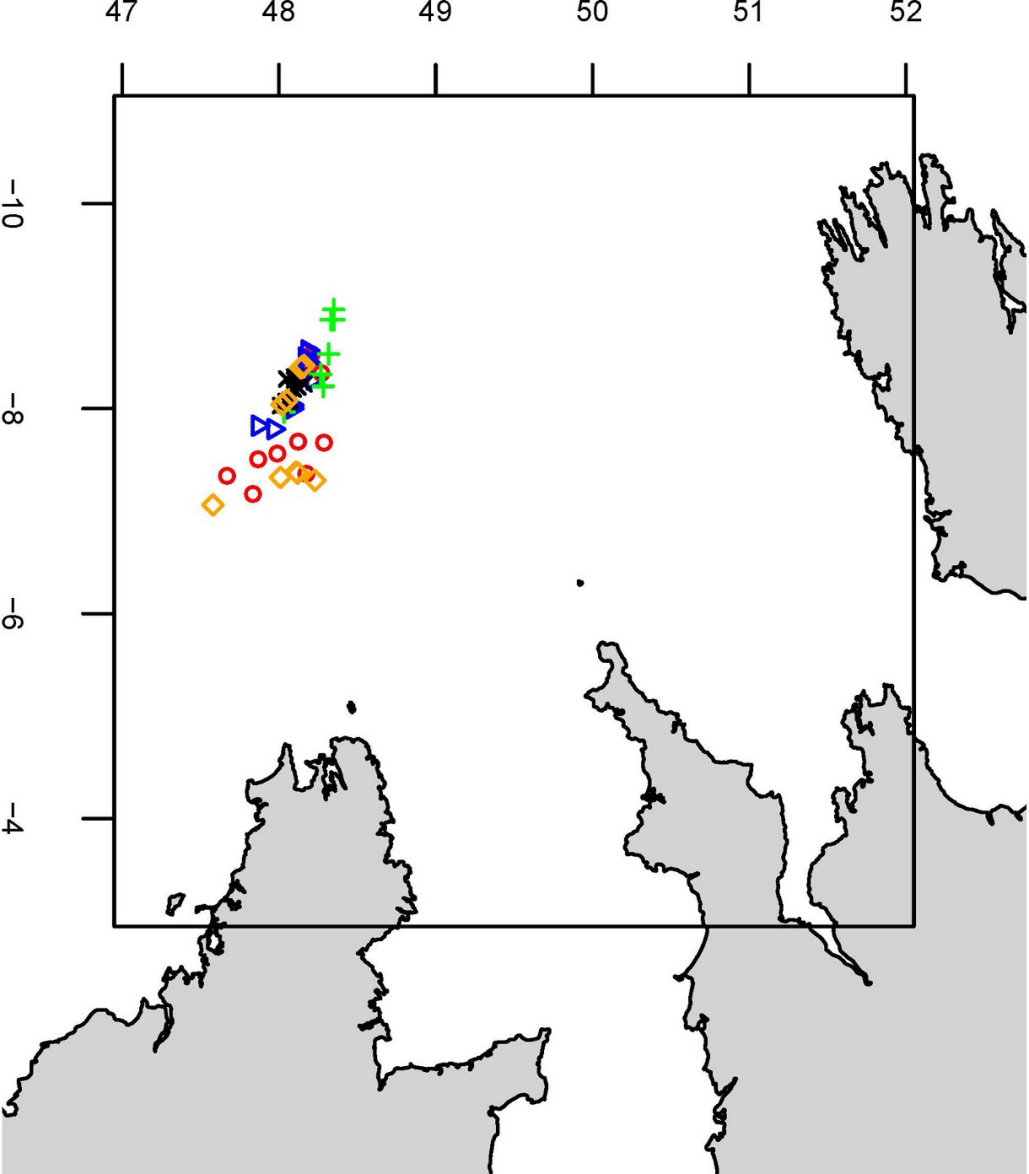
Map of the study showing the location of the 49 hauls sampled. The different symbols illustrate the four trips.

Five fishing trips were conducted between September 2014 and October 2015 in the southern part of the Celtic sea (Fig 2). Mean (± standard deviation) tow duration was 3.4 (±0.2) h, and mean fishing depth was 219 (±108 m (S1 Table)). For 49 hauls, wanted catches and unwanted catches (as usually sorted by the crew) were weighed (kg) per species, and the length of individual fish was measured (to the nearest 1 cm). When the total catch was too large to allow measurement of every individual, random sub-sampling was performed and the weight ratio between total catch and the subsample was used to raise the data. Due to identification issues, data for certain individual species were combined into taxonomic groups: *Lophius* spp (*Lophius piscatorius* and *L budegassa*), *Loligo* spp *(Loligo forbesii* and *L*. *vulgaris*), rays, gurnard and *Trisopterus* spp (*Trisopterus luscus*, *T*. *minutus* and *T*. *esmarkii*).

Length-dependent relative size selectivity between the experimental and control trawls was analyzed. Logistic regression in the mixed generalized linear model framework was fitted for 6 species according to the methods used by Holst and Revill [19] and Vogel et al. [20]. Between haul variability was incorporated by testing the significance of fixed effects: depth, tow duration and catch. Including haul as random effect, allowed accounting for an additional between haul variability, not explained by the fixed effects, and which can be interpreted as the effect of environmental and/or external conditions (e.g. current, time of day, fish behavior and physiology, fish species composition, and season) on catches. The within haul variability was taken into account at the individual level, with the effect of size on retention probability. For each species, observations outside the 0.025 and 0.975 quantiles of length distribution (which represents 5% of the data set) were excluded to reduce the influence of outliers on the fits. The proportion of fish retained per length class, P(l), were calculated and modeled as:

$$\begin{array}{l} Logit\;\left(P_{h}(l\right))=logit\;\left(\frac{\;N_{t,h}\left(l\right)}{N_{t,h}\;\left(l\right)\;+N_{c,h}\;\left(l\right)}\right)\\ \;=\beta_{0}+\overset{m}{\underset{i=1}{\sum }}\beta_{i}l^{i}+\beta_{5}W_{h}+\;\beta_{6}TD_{h}+\beta_{7}D_{h}\;+S_{h}+\;\delta_{h},\;with\;\delta_{h}\;\sim N\left(0,\sigma ²\right) \end{array}$$

where *N*_*t*,*h*_ (*l*) is the number of fish of length l in the test trawl; *N*_*c*,*h*_ (*l*) is the number of fish of length l in the control trawl; m is the degree of polynomial function that models the effect of size (m = 0–3); *W*_*h*_ total catch weigh in kg, *TD*_*h*_ the tow duration in hour, *D*_*h*_ the fishing depth in meter, *S*_*h*_ the subsampling ratio and *δ*_h_ is the random effect on the haul. The subsampling ratio is modeled as an offset [19]: $S_{h}=\;log(\frac{\;q_{t}\left(h\right)}{q_{c}\;\left(h\right)})$ with *q*_*t*_ (*h*) and *q*_*c*_ (*h*) the proportions taken out for measurements from the catch bulk of the experimental and control gear, respectively. The minimal dataset is provided as a supporting information file.

For each species, the best-fitted model was selected based on the Akaike information criterion (AIC) considering improvement between two models to be significant when differences between AIC values exceeded 5. When two models had similarly “good” AIC values (i.e. Δ_AIC_ < 5) the most parsimonious one was selected. The 32 models tested are summarized in Table 1. Statistical analyses were performed using R 3.5.3 [21].

**Table 1:** Description of the model from the null to the full model (M1 to M32).

Model	Length	Length²	Length^3^	WEIGHT	FISHING_DURATION	DEPTH	df
M1							2
M2	**×**						3
M3		**×**					4
M4			**×**				5
M5				**×**			3
M6	**×**			**×**			4
M7		**×**		**×**			5
M8			**×**	**×**			6
M9					**×**		3
M10	**×**				**×**		4
M11		**×**			**×**		5
M12			**×**		**×**		6
M13						**×**	3
M14	**×**					**×**	4
M15		**×**				**×**	5
M16			**×**			**×**	6
M17					**×**	**×**	4
M18	**×**				**×**	**×**	5
M19		**×**			**×**	**×**	6
M20			**×**		**×**	**×**	7
M21				**×**		**×**	4
M22	**×**			**×**		**×**	5
M23		**×**		**×**		**×**	6
M24			**×**	**×**		**×**	7
M25				**×**	**×**		4
M26	**×**			**×**	**×**		5
M27		**×**		**×**	**×**		6
M28			**×**	**×**	**×**		7
M29				**×**	**×**	**×**	5
M30	**×**			**×**	**×**	**×**	6
M31		**×**		**×**	**×**	**×**	7
M32			**×**	**×**	**×**	**×**	8

All model included haul as random effect and subsampling as an offset. The superscripts refer to the degree of polynomial function that models the effect of size.

### Ethics statement

Sea trials were carried out on board the vessel An Triskell in accordance with the European scientific fishing authorization granted by the French maritime fisheries and aquaculture directorate (n.o 2014/730412/SELECMCTrawl/0006 and 2015/730412/SELECMCTrawl/0001). Animals were not exposed to additional stress other than that involved in commercial fishing practice. Thus, no additional authorization or ethics approval was required to perform the study. This study did not involve endangered or protected species.

## Results

### Target species

#### Lepidorhombus whiffiagonis

**Fig 3:**
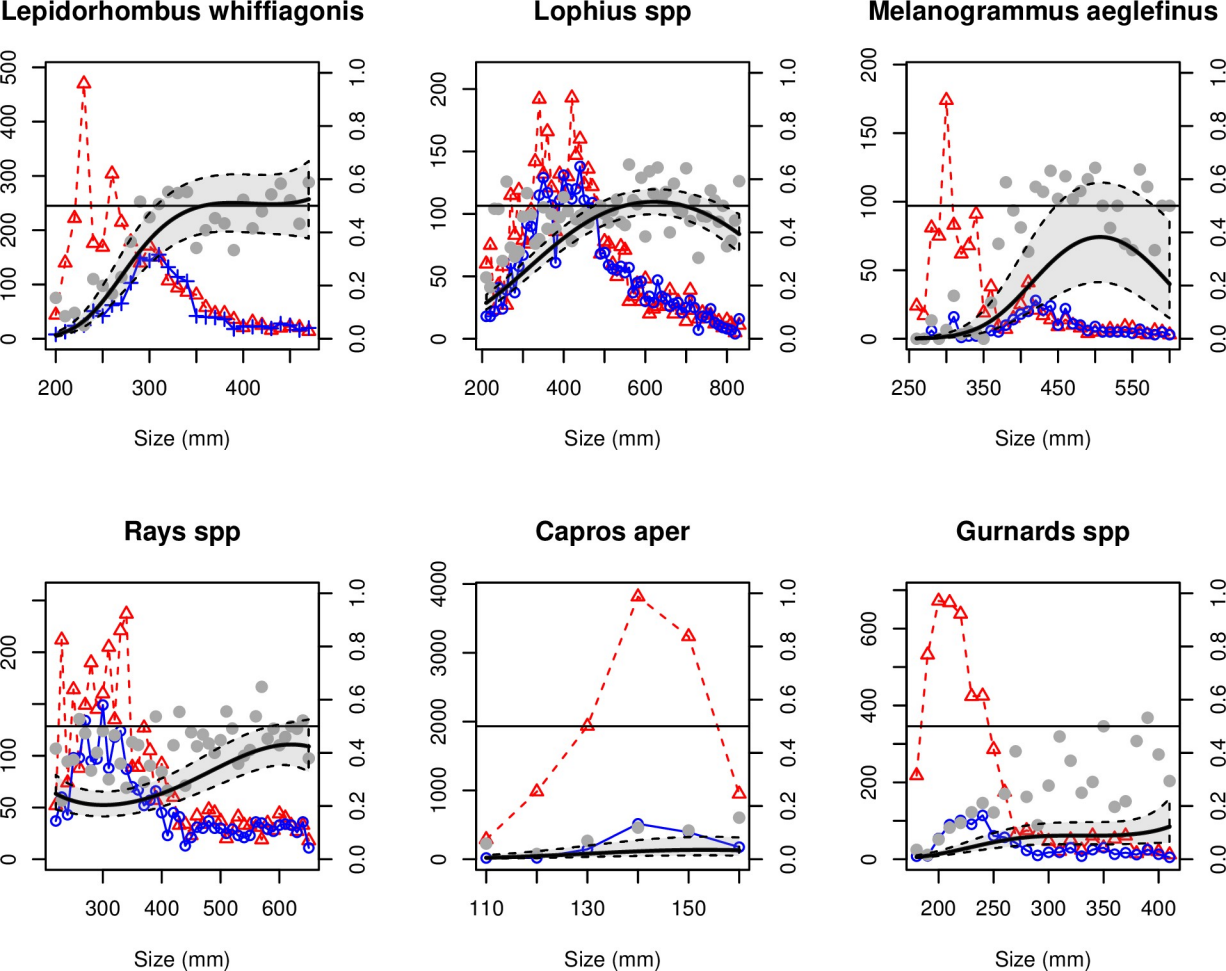
Analyses of size data. For each taxonomic group: Number of fish caught as a function of length in the control trawl (dashed red line and triangles) and test trawl (solid blue line and circles) (left axes) and mean proportions retained (gray circles), with the margin prediction of retention at length for the best-fitted model (black line) and its associated 95% confidence interval (black dashed polygon) (right axes). The other covariates were set to their means when making the predictions.

The best-fitted model (Table 2) indicated that megrim shorter than the MCRS (20 cm) were nearly absent from both trawls, those 20–30 cm long were retained more by the control than the test trawl and those longer than 30 cm had an equal retention probability for the two trawls (Fig 3). None of the covariables tested were retained during the model selection procedure (Table 2).

**Table 2:** Akaike information criterion (AIC) values of the models fitted for each species.

Rays spp		Melanogrammus aeglefinus		Lophius		Lepidorhombus whiffiagonis		Gurnards spp		Capros aper	
Model	AIC	Model	AIC	Model	AIC	Model	AIC	Model	AIC	Model	AIC
**M12**	**4842.5**	M19	780.3	M12	7111.4	**M4**	**3596.4**	**M28**	**2357.8**	M12	1141.8
M28	4842.6	M20	781.8	**M11**	**7111.4**	M16	3596.6	M32	2358.5	M11	1142.3
M20	4842.7	M31	782.4	M20	7111.4	M12	3597.7	M12	2363.5	M28	1143.9
M32	4842.9	**M15**	**783.0**	M19	7111.5	M20	3597.7	M20	2364.7	M20	1143.9
M27	4849.9	M32	783.9	M27	7112.3	M24	3598.1	M8	2364.8	M27	1144.4
M11	4850.0	M16	784.3	M28	7112.3	M8	3598.2	M24	2365.4	M19	1144.5
M31	4850.0	M23	784.8	M32	7112.6	M32	3598.8	M27	2365.5	M4	1144.6
M19	4850.1	M24	786.1	M31	7112.6	M28	3599.3	M31	2366.2	**M3**	**1145.4**
M4	4850.8	M11	789.5	M4	7126.2	M3	3607.9	M4	2367.8	M32	1146.0
M16	4851.3	M12	790.9	M3	7126.2	M15	3608.2	M16	2368.9	M31	1146.6
M8	4851.9	M27	791.4	M16	7126.5	M11	3609.1	M11	2371.3	M8	1146.6
M24	4852.4	M3	791.9	M15	7126.5	M19	3609.3	M19	2372.5	M16	1146.8
M3	4858.0	M28	792.9	M7	7127.9	M7	3609.6	M7	2372.7	M7	1147.3
M10	4858.2	M4	793.2	M8	7127.9	M23	3609.7	M23	2373.5	M15	1147.5
M26	4858.3	M7	793.6	M24	7128.3	M31	3610.4	M3	2375.9	M24	1148.8
M15	4858.3	M8	794.9	M23	7128.4	M27	3610.7	M15	2377.0	M23	1149.5
M18	4858.5	M18	817.5	M10	7209.7	M2	3775.0	M26	2412.1	M10	1158.3
M30	4858.6	M14	819.0	M26	7210.4	M14	3775.9	M30	2413.1	M26	1160.4
M7	4859.0	M30	819.6	M18	7210.9	M10	3776.4	M10	2418.4	M18	1160.4
M23	4859.4	M22	821.0	M30	7211.8	M6	3776.5	M18	2419.9	M2	1161.6
M2	4865.7	M10	825.2	M2	7225.4	M22	3776.9	M6	2420.4	M30	1162.5
M14	4866.3	M2	826.7	M14	7226.7	M18	3777.0	M22	2421.4	M6	1163.5
M6	4866.8	M26	827.3	M6	7227.1	M26	3777.6	M2	2423.8	M14	1163.7
M22	4867.4	M6	828.7	M22	7228.4	M30	3777.7	M14	2425.1	M22	1165.6
M29	4936.5	M17	977.3	M9	7451.8	M29	4273.8	M25	2557.5	M9	1202.3
M25	4937.0	M13	979.0	M25	7452.1	M17	4274.8	M29	2558.4	M25	1204.3
M17	4937.7	M29	979.3	M17	7452.7	M13	4274.8	M9	2565.7	M17	1204.4
M9	4938.3	M21	981.0	M29	7453.2	M21	4274.8	M5	2565.9	M1	1205.8
M13	4946.5	M9	984.4	M1	7469.3	M1	4275.8	M21	2566.8	M29	1206.4
M1	4946.6	M1	986.2	M13	7470.2	M9	4276.3	M17	2567.1	M5	1207.6
M21	4946.8	M25	986.5	M5	7470.8	M25	4276.6	M1	2570.7	M13	1207.8
M5	4946.9	M5	988.2	M21	7471.8	M5	4276.7	M13	2572.0	M21	1209.7

Selected models are in bold.

#### Lophius spp

Analysis of length showed that equal retention probability was reached at ca. 40 cm (Fig 3) and that the difference in the retention probability between the two trawls was small for shorter fish. Tow duration had significant effect (Table 2, p-value <0.001), with in the proportion of anglerfish retained per length class decreasing with increasing tow duration.

#### Melanogrammus aeglefinus

The best-fitted model (Table 2) indicated that haddock shorter than 30–35 cm, while present in the control trawl, were nearly absent from the test trawl, and the size at which retention probability was equal was ca. 40–45 cm (Fig 3). This size is much higher than the MCRS of 30 cm. Fishing depth was retained in model selection procedure (Table 2, p-value<0.001) indicating that proportion of haddock retained per length class increased with increasing depth.

### Rays

Despite high variability in the dataset, the best-fitted model (Table 2) tended to indicate a slightly lower retention probability for test trawl than the control trawl for rays shorter than 45 cm (Fig 3). The proportion of rays retained per length class decreased with increasing tow duration (Table 2, p-value<0.001).

### Non-target species

#### Capros aper

Boarfish 11–16 cm long, abundant in the control trawl, were nearly absent in the test trawl (Fig 3). None of the covariables measured affected the retention probability of boarfish (Table 2).

#### Gurnard

Length analysis showed a higher retention probability for individuals shorter than 25–27 cm for the control trawl. The best-fitted model (Table 2) had difficulty estimating the size at which retention probability was equal since the control trawl seemed to have systematically more gurnard 30–40 cm long (Fig 3). Tow duration and total catch weight were retained in model selection procedure (p-value = 0.002 and p-value = 0.003, respectively) indicating that the proportion of gurnard retained per length class decreased with increasing tow duration and catch weight.

## Discussion

To the best of our knowledge, this was the first time a 100 mm T90 mesh configuration was tested simultaneously in the extension and codend, although T90 mesh has been individually tested in the codend [10, 16, 22] or the extension [14]. Given the previous results, the device tested in this study is interesting in terms of shape, length, mesh type and mesh size for the dermersal mixed fisheries in the Celtic Sea. The device aims to reduce, as much as possible, catches of target species below the MCRS and all sizes of non-target species. In practice, it should drastically decrease the overall amount of unwanted catches that fishers previously discarded and should now store on-board and land in port to comply with the new common fishery policy regulation.

T90 mesh is known to increase the escape rate of a wide range of species, from flatfish to roundfish and from demersal to pelagic species [10–16], which is particularly useful for mixed fisheries. T90 mesh remains open while towing and, for a given stretched size, provides a larger space for fish to escape than diamond mesh. Compared to the mandatory upper panel designed to enhance escapement of species that exhibit “rising” escapement behavior, the use of a cylinder provides an escape area on the top, sides and the bottom of the gear. This should benefit a wider range of species that have other behaviors and escape strategies such as sinking or horizontal escapement behavior. At more than 160 mesh and 12.5 m long, the selective cylinder is longer than those used in other trials with a T90 extension and codend. Additionally, as explained by Kopp et al. [14] and Digre et al. [22], a selective panel mounted on the extension is designed to increase an escapee’s survival potential by not damaging it and allowing it to avoid compression in the codend.

In this study, the use of T90 mesh increased the size selectivity of commonly encountered roundfish, as previously highlighted for beam trawling [16] and bottom trawling [13]. Our results confirm previous results for haddock and extend them to other species such as boarfish, gurnard, megrim, monkfish and rays. Catches of haddock below the MCRS were reduced to nearly zero (S3 Table). However, the size at which the retention probability was equal between the two gears was estimated to be much higher than the MCRS, which would increased non-negligible commercial losses. By extension, even more individuals of other gadoid species, such as whiting and hake, with a thinner cross-section than haddock for a given length, would escape through this device. For fleets that depend on small-grade haddock and whiting, the 100 mm T90 extension and codend device could result in commercial losses that are too large to remain profitable. In such cases, decreasing the mesh size [23] or mounting the T90 only in the extension or codend could be useful alternatives.

Gurnard and boarfish, non-target species that could be caught in large quantities in a single haul, have coarse scales and spines that damage other species in the trawl. Fishers are thus greatly interested in decreasing gurnard and boarfish catches. More generally, the fishing master and crew report greatly improved quality in the catch when using T90 gear, which is an additional benefit [22, 24]. Interestingly, even though the TAC of boarfish exceeds 100 000 t, France has zero quota for this species. Thus, the landing obligation will cause serious issues for French fishers, as this non-target species could choke the entire whitefish directed demersal fisheries. One important result is that the device tested almost eliminated capture of boarfish. The shape of T90 mesh perfectly suited the shape of boarfish, probably facilitating the its escape. Boarfish is usually captured in large schools that tend to obstruct the codend. The large surface area of the T90 mesh increased the probability that the entire school would escape from the extension and the codend. The few Atlantic horse mackerel (*Trachurus trachurus*) observed were not representative of large catches. However, our results are consistent with those of Kopp et al. [14], who reported the strong ability of T90 mesh to eliminate unwanted catches of small and medium pelagic species, for which demersal fleets have a low or zero quota at the vessel level.

Small megrim (smaller than MCRS of 20 cm) were rarely captured, even with the control trawl, but it was not possible to determine from our dataset whether small megrim escaped from the control trawl or whether they were not present in the environment. Only an absolute size selectivity experiment could address this issue. Our result showed a major reduction in marketable megrim of 20–30 cm long. Kopp et al. [14] observed increased selectivity for sole (*Solea solea*) using a 100 mm T90 extension. Mean target catch weights of other flatfish, such as *M*. *kitt* were similar between the two trawls, which differs from the results of Bayse et al. [16], who used beam trawls and found that a T90 codend decreased selectivity for flatfish. Similarly, Lomeli et al. [13] suggested that, compared to diamond mesh, T90 mesh decreased the 50% retention length for flatfish. Relative selectivity for monkfish and rays increased slightly, however, resulting in a significant decrease in unwanted catch weights for rays but not for monkfish. Our results rely on a large dataset collected during an entire year to assess impacts on the largest number of species possible. They highlight that the device should not be used during seasonal trips that target red mullet or cephalopods. Increasing size selectivity for red mullet in the Mediterranean Sea by using a smaller T90 mesh has been previously observed [12].

The effect of catch size on codend selectivity has been addressed in numerous studies, with conflicting results [25–28]. Indeed, the underlying process appears nonlinear, while moderate catch might increase size opening and enhance escapement in the codend, very large catch sizes can obstruct the codend meshes and thereby reduce the potential escape for fish. The effect of catch weight on catch at length was not retained by model selection for the 6 species studied. The only species for which catch weigh may have a small impact is gurnard, with a significant negative effect of total catch. Other environment conditions such as tow duration and fishing depth were incorporated in the models. The hypothesis that catch is proportional to tow duration has been tested in several studies [29, 30]. Negative effect of tow duration on different phases of the haul (towing, hand up, surface) have already been highlighted for haddock, whiting and Norway lobster by Madsen et al. [31]. In this analysis, tow duration seems to decrease the retention probability at length for rays, monkfish and gurnards. Differences observed are often associated with size-related swimming capacities, with larger fish having a greater swimming capacity than small fish [32]. Time of the day (day/night), season or fish condition, has non negligible impacts on the effectiveness of the test gear. The sampling design did not allow us to formally assess their respective effect; however, this remaining variability was handled by implementing “haul” as a random term in the modeling process.

The experimental fishing gear evaluated in this study will perform well at a commercial level when targeting monkfish, megrim, rays and large haddock, however fishers are not likely to use this gear when targeting smaller-bodied species such as cephalopods, small haddock, whiting and hake. Selectivity studies often focus on a short list of target species; however, catches of non-target species under TAC and for which quota availability is small (or even null) can be problematic for some fisheries. The experimental fishing gear constitutes an efficient solution to mitigate catches non-target schooling fish such as boarfish.

## Supporting information

S1 Table Haul characteristics in mean depth, duration, quarter and total catch weight. (DOCX)

S2 Table Catch characteristic per haul. (DOCX)

S3 Table Mean observed weight (in kg) per species in the wanted catches (LAN) and unwanted catches (DIS). (DOCX)

S1 Dataset (CSV)

## References

[1] EU. Regulation (EU) No 1380/2013 of the European Parliament and of the Council of 11 December 2013 on the Common Fisheries Policy, amending Council Regulations (EC) No 1954/2003 and (EC) No 1224/2009 and repealing Council Regulations (EC) No 2371/2002 and (EC) No 639/2004 and Council Decision 2004/585/EC. 2013. Available: https://eur-lex.europa.eu/eli/reg/2013/1380/oj. Accessed 19 Sep 2018.

[2] Sarda F, Coll M, Heymans JJ, Stergiou KI. Overlooked impacts and challenges of the new European discard ban. Fish Fish. 2015;16: 175–180. 10.1111/faf.12060

[3] Herrmann B, Wienbeck H, Moderhak W, Stepputtis D, Krag LA. The influence of twine thickness, twine number and netting orientation on codend selectivity. Fish Res. 2013;145: 22–36. 10.1016/j.fishres.2013.03.002

[4] MacLennan DN. Fishing gear selectivity: an overview. Fish Res. 1992;13: 201–204. 10.1016/0165-7836(92)90076-6

[5] O’Neill FG, Mutch K. Selectivity in Trawl Fishing Gears. Scott Mar Freshw Sci. 2017; Vol 8 No 01.

[6] Feekings J, Bartolino V, Madsen N, Catchpole T. Fishery Discards: Factors Affecting Their Variability within a Demersal Trawl Fishery. Plos One. 2012;7: e36409 10.1371/journal.pone.0036409 22558463 PMC3340337

[7] Sajdlova Z, Drastik V, Juza T, Riha M, Frouzova J, Cech M, et al Fish behaviour in response to a midwater trawl footrope in temperate reservoirs. Fish Res. 2015;172: 105–113. 10.1016/j.fishres.2015.06.025

[8] Krag LA, Herrmann B, Karlsen JD. Inferring Fish Escape Behaviour in Trawls Based on Catch Comparison Data: Model Development and Evaluation Based on Data from Skagerrak, Denmark (vol 9, e88819, 2014). Plos One. 2014;9: e100605 10.1371/journal.pone.0100605 PMC3930632 24586403

[9] Ryer CH. A review of flatfish behavior relative to trawls. Fish Res. 2008;90: 138–146. 10.1016/j.fishres.2007.10.005

[10] Herrmann B, Priour D, Krag LA. Simulation-based study of the combined effect on cod-end size selection of turning meshes by 90 degrees and reducing the number of meshes in the circumference for round fish. Fish Res. 2007;84: 222–232. 10.1016/j.fishres.2006.10.020

[11] Deval MC, Ozgen G, Ozbilgin H. Selectivity of 50mm T0 and T90 codends for commercial shrimp species in the Turkish deepwater trawl fishery, Eastern Mediterranean. J Appl Ichthyol. 2016;32: 1041–1057. 10.1111/jai.13128

[12] Tokac A, Herrmann B, Aydin C, Kaykac H, Unluler A, Gokce G. Predictive models and comparison of the selectivity of standard (TO) and turned mesh (T90) codends for three species in the Eastern Mediterranean. Fish Res. 2014;150: 76–88. 10.1016/j.fishres.2013.10.015

[13] Lomeli MJM, Hamel OS, Wakefield WW, Erickson DL. Improving Catch Utilization in the US West Coast Groundfish Bottom Trawl Fishery: an Evaluation of T90-Mesh and Diamond-Mesh Cod Ends. Mar Coast Fish. 2017;9: 149–160. 10.1080/19425120.2016.1274697

[14] Kopp D, Morandeau F, Mouchet M, Vogel C, Mehault S. What can be expected of a T90 extension piece to improve selectivity in bottom trawl multispecific fisheries in the Bay of Biscay? Fish Sci. 2018;84: 597–604. 10.1007/s12562-018-1203-8

[15] Madsen N, Herrmann B, Frandsen RP, Krag LA. Comparing selectivity of a standard and turned mesh T90 codend during towing and haul-back. Aquat Living Resour. 2012;25: 231–240. 10.1051/alr/2012021

[16] Bayse SM, Herrmann B, Lenoir H, Depestele J, Polet H, Vanderperren E, et al Could a T90 mesh codend improve selectivity in the Belgian beam trawl fishery? Fish Res. 2016;174: 201–209. 10.1016/j.fishres.2015.10.012

[17] EU. Commision delegated regulation (EU) 2018/ 47 of 30 October 2017—authorising the use of alternative T90 trawls in Baltic Sea fisheries, by way of derogation from Council Regulation (EC) No 2187 / 2005. 2018; 2.

[18] Cornou A-S, Goascoz N, Scavinner M, Chassanite A, Dubroca L, Rochet M-J. Captures et rejets des métiers de pêche français. Résultats des observations à bord des navires de pêche professionnelle en 2016. 2017 Available: http://archimer.ifremer.fr/doc/00353/46441/

[19] Holst R, Revill A. A simple statistical method for catch comparison studies. Fish Res. 2009;95: 254–259. 10.1016/j.fishres.2008.09.027

[20] Vogel C, Kopp D, Morandeau F, Morfin M, Mehault S. Improving gear selectivity of whiting (Merlangius merlangus) on board French demersal trawlers in the English Channel and North Sea. Fish Res. 2017;193: 207–216. 10.1016/j.fishres.2017.04.013

[21] R Core Team. R: A Language and Environment for Statistical Computing. https://www.R-project.org/. 2017.

[22] Digre H, Hansen UJ, Erikson U. Effect of trawling with traditional and “T90” trawl codends on fish size and on different quality parameters of cod Gadus morhua and haddock Melanogrammus aeglefinus. Fish Sci. 2010;76: 549–559. 10.1007/s12562-010-0254-2

[23] Browne D, Cosgrove R, Tyndall P. Assessment of T90 mesh in a fishery targeting whiting in the Celtic Sea. Irish Sea Fisheries Board (BIM); 2016 p. 8 Available: http://www.bim.ie/media/bim/content/publications/5536,BIM,Assessment,-,T90,mesh,-,Whiting,-,Celtic,Sea,-,ONLINE.pdf

[24] Lamothe J, Larnaud P, Fiche M, Robert M, Morandeau F, Vacherot J-P, et al Projet CELSELEC. Amélioration de la sélectivité des chalutiers hauturiers en mer Celtique. 2017 Available: 10.13155/51488

[25] Erickson DL, Perez-Comas JA, Pikitch EK, Wallace JR. Effects of catch size and codend type on the escapement of walleye pollock (Theragra chalcogramma) from pelagic trawls. Fish Res. 1996;28: 179–196. 10.1016/0165-7836(96)00497-3

[26] Herrmann B. Effect of catch size and shape on the selectivity of diamond mesh cod-ends: II. Theoretical study of haddock selection. Fish Res. 2005;71: 15–26. 10.1016/j.fishres.2004.08.021

[27] O’Neill FG, Kynoch RJ. The effect of cover mesh size and cod-end catch size on cod-end selectivity. Fish Res. 1996;28: 291–303. 10.1016/0165-7836(96)00501-2

[28] Sala A, Herrmann B, De Carlo F, Lucchetti A, Brcic J. Effect of codend circumference on the size selection of square-mesh codends in trawl fisheries. Plos One. 2016;11: e0160354 10.1371/journal.pone.0160354 27472058 PMC4966963

[29] Godø OR, Pennington M, Vølstad JH. Effect of tow duration on length composition of trawl catches. Fish Res. 1990;9: 165–179. 10.1016/0165-7836(90)90062-Z

[30] Sala A. Influence of tow duration on catch performance of trawl survey in the Mediterranean Sea. Plos One. 2018; e0191662 10.1371/journal.pone.0191662 29357381 PMC5777655

[31] Madsen N, Skeide R, Breen M, Krag LA, Huse I, Soldal AV. Selectivity in a trawl codend during haul-back operation—An overlooked phenomenon. Fish Res. 2008;91: 168–174. 10.1016/j.fishres.2007.11.016

[32] He P. Behavior of Marine Fishes: Capture Processes and Conservation Challenges. Blackwell Publishing 2010.

