# Supplementary material for: Toward elimination of unwanted catches using a 100 mm T90 extension and codend in demersal mixed fisheries
Source: PLoS One. 2020 Jul 8;15(7):e0235368. doi: 10.1371/journal.pone.0235368 (PMC7343172; doi:10.1371/journal.pone.0235368)
Supplement: S1 Table — (DOCX) [file pone.0235368.s001.docx]

Supporting Information

S1 Table. Haul characteristics in mean depth, duration, quarter and total catch weight.

| Haul ID | Depth (m) | Haul_Duration (h) | Quarter | Catch_weight (kg) |
| --- | --- | --- | --- | --- |
| 10410912_10 | 176 | 3.3 | 4 | 355 |
| 10410912_14 | 173 | 3.3 | 4 | 313 |
| 10410912_18 | 172 | 3.15 | 4 | 313 |
| 10410912_2 | 173 | 3.9 | 4 | 265 |
| 10410912_21 | 180 | 3.3 | 4 | 353 |
| 10410912_29 | 191 | 3.3 | 4 | 286 |
| 10410912_6 | 156 | 3.3 | 4 | 447 |
| 8962643_13 | 181 | 3.43 | 3 | 392 |
| 8962643_2 | 275 | 3.6 | 3 | 484 |
| 8962643_23 | 159 | 3.3 | 3 | 396 |
| 8962643_27 | 173 | 3 | 3 | 446 |
| 8962643_31 | 169 | 3.3 | 3 | 542 |
| 8962643_35 | 168 | 3.3 | 3 | 499 |
| 8962643_38 | 169 | 3.31 | 3 | 443 |
| 8962643_42 | 175 | 3.3 | 3 | 462 |
| 8962643_47 | 198 | 3.75 | 3 | 427 |
| 8962643_5 | 380 | 3.3 | 3 | 281 |
| 8962643_9 | 188 | 3.3 | 3 | 446 |
| 8963109_14 | 343 | 3.3 | 3 | 452 |
| 8963109_18 | 360 | 3.3 | 3 | 409 |
| 8963109_2 | 226 | 3.35 | 3 | 561 |
| 8963109_23 | 558 | 3.3 | 3 | 427 |
| 8963109_27 | 499 | 3.3 | 3 | 448 |
| 8963109_33 | 643 | 3.3 | 3 | 238 |
| 8963109_4 | 239 | 3.4 | 3 | 376 |
| 8963109_45 | 184 | 3.3 | 3 | 518 |
| 8963109_6 | 210 | 3.4 | 3 | 532 |
| 8983948_14 | 175 | 2.85 | 1 | 339 |
| 8983948_15 | 184 | 3.6 | 1 | 324 |
| 8983948_18 | 166 | 3.3 | 1 | 548 |
| 8983948_22 | 178 | 3.3 | 1 | 435 |
| 8983948_23 | 178 | 3.3 | 1 | 472 |
| 8983948_26 | 178 | 3.6 | 1 | 429 |
| 8983948_27 | 177 | 3.3 | 1 | 505 |
| 8983948_37 | 164 | 3.9 | 1 | 244 |
| 8983948_38 | 169 | 3.3 | 1 | 451 |
| 8983948_40 | 175 | 4.2 | 1 | 467 |
| 8983948_7 | 165 | 3.15 | 1 | 274 |
| 8983948_8 | 137 | 3.3 | 1 | 258 |
| 9144311_10 | 186 | 3.3 | 2 | 473 |
| 9144311_14 | 179 | 3.3 | 2 | 459 |
| 9144311_19 | 215 | 3.3 | 2 | 526 |
| 9144311_26 | 178 | 3.3 | 2 | 514 |
| 9144311_27 | 178 | 3.3 | 2 | 419 |
| 9144311_3 | 199 | 3.15 | 2 | 365 |
| 9144311_30 | 175 | 3.15 | 2 | 475 |
| 9144311_34 | 180 | 3.3 | 2 | 455 |
| 9144311_38 | 199 | 3.15 | 2 | 425 |
| 9144311_42 | 265 | 3.45 | 2 | 481 |
| mean | 219 | 3.35 |  | 417 |
| sd | 104 | 0.22 |  | 87 |
