# Supplementary material for: Toward elimination of unwanted catches using a 100 mm T90 extension and codend in demersal mixed fisheries
Source: PLoS One. 2020 Jul 8;15(7):e0235368. doi: 10.1371/journal.pone.0235368 (PMC7343172; doi:10.1371/journal.pone.0235368)
Supplement: S2 Table — (DOCX) [file pone.0235368.s002.docx]

S2 Table. Catch characteristic per haul

|  |  | Control gear | | Test gear | |
| --- | --- | --- | --- | --- | --- |
| Haul ID | Species | Total Nbr | Mean Subsampling | Total Nbr | Mean Subsampling |
| 10410912_14 | Capros aper | 6 | 0.5 | 3 | 0.33 |
| 10410912_18 |  | 2 | 0.5 | 0 | 1 |
| 10410912_2 |  | 14 | 0.35 | 22 | 0.65 |
| 10410912_21 |  | 121 | 0.27 | 31 | 0.39 |
| 10410912_29 |  | 68 | 0.52 | 6 | 0.67 |
| 10410912_6 |  | 1132 | 0.03 | 212 | 0.14 |
| 8962643_13 |  | 93 | 0.15 | 0 | 1 |
| 8962643_2 |  | 112 | 0.13 | 18 | 0.17 |
| 8962643_23 |  | 416 | 0.14 | 25 | 0.35 |
| 8962643_27 |  | 28 | 0.21 | 36 | 0.33 |
| 8962643_31 |  | 6 | 0.17 | 18 | 0.33 |
| 8962643_35 |  | 16 | 0.13 | 10 | 0.31 |
| 8962643_38 |  | 347 | 0.15 | 10 | 0.4 |
| 8962643_42 |  | 451 | 0.13 | 30 | 0.33 |
| 8962643_47 |  | 84 | 0.12 | 0 | 1 |
| 8962643_5 |  | 41 | 1 | 2 | 1 |
| 8962643_9 |  | 746 | 0.04 | 18 | 0.17 |
| 8963109_14 |  | 67 | 0.06 | 8 | 0.13 |
| 8963109_18 |  | 39 | 0.08 | 7 | 0.14 |
| 8963109_23 |  | 580 | 0.1 | 102 | 0.17 |
| 8963109_27 |  | 646 | 0.05 | 85 | 0.2 |
| 8963109_33 |  | 8 | 0.13 | 0 | 1 |
| 8963109_4 |  | 47 | 0.08 | 9 | 0.11 |
| 8963109_45 |  | 905 | 0.04 | 81 | 0.18 |
| 8963109_6 |  | 13 | 0.08 | 9 | 0.11 |
| 8983948_14 |  | 199 | 0.07 | 20 | 0.2 |
| 8983948_15 |  | 70 | 0.09 | 0 | 1 |
| 8983948_18 |  | 79 | 0.09 | 0 | 1 |
| 8983948_22 |  | 99 | 0.12 | 35 | 0.24 |
| 8983948_23 |  | 165 | 0.11 | 16 | 0.25 |
| 8983948_26 |  | 0 | 1 | 10 | 0.2 |
| 8983948_37 |  | 41 | 0.14 | 0 | 1 |
| 8983948_38 |  | 28 | 0.14 | 24 | 0.25 |
| 8983948_40 |  | 1742 | 0.01 | 0 | 1 |
| 8983948_7 |  | 14 | 0.21 | 5 | 0.6 |
| 8983948_8 |  | 18 | 0.11 | 2 | 1 |
| 9144311_10 |  | 759 | 0.04 | 16 | 0.37 |
| 9144311_14 |  | 115 | 0.25 | 11 | 0.47 |
| 9144311_19 |  | 15 | 0.2 | 6 | 0.33 |
| 9144311_26 |  | 216 | 0.17 | 9 | 0.54 |
| 9144311_3 |  | 33 | 0.21 | 2 | 0.5 |
| 9144311_30 |  | 23 | 0.21 | 0 | 1 |
| 9144311_34 |  | 12 | 0.25 | 0 | 1 |
| 9144311_38 |  | 131 | 0.24 | 49 | 0.38 |
| 9144311_42 |  | 1459 | 0.02 | 318 | 0.09 |
| 10410912_10 | Gurnards spp | 90 | 0.25 | 37 | 0.45 |
| 10410912_14 |  | 104 | 0.46 | 137 | 0.39 |
| 10410912_18 |  | 57 | 0.51 | 61 | 0.53 |
| 10410912_2 |  | 28 | 0.35 | 28 | 0.56 |
| 10410912_21 |  | 129 | 0.26 | 64 | 0.36 |
| 10410912_29 |  | 57 | 0.56 | 2 | 1 |
| 10410912_6 |  | 76 | 0.25 | 20 | 0.5 |
| 8962643_13 |  | 134 | 0.15 | 10 | 0.2 |
| 8962643_2 |  | 40 | 0.13 | 0 | 1 |
| 8962643_23 |  | 42 | 0.14 | 3 | 0.33 |
| 8962643_27 |  | 79 | 0.21 | 9 | 0.33 |
| 8962643_31 |  | 147 | 0.16 | 6 | 0.33 |
| 8962643_35 |  | 155 | 0.13 | 3 | 0.33 |
| 8962643_38 |  | 86 | 0.15 | 7 | 0.45 |
| 8962643_42 |  | 99 | 0.13 | 9 | 0.33 |
| 8962643_47 |  | 225 | 0.12 | 0 | 1 |
| 8962643_5 |  | 26 | 1 | 2 | 1 |
| 8962643_9 |  | 162 | 0.39 | 57 | 0.42 |
| 8963109_14 |  | 305 | 0.16 | 58 | 0.22 |
| 8963109_18 |  | 143 | 0.23 | 16 | 0.29 |
| 8963109_2 |  | 74 | 0.04 | 0 | 1 |
| 8963109_23 |  | 140 | 0.19 | 74 | 0.25 |
| 8963109_27 |  | 132 | 0.18 | 31 | 0.25 |
| 8963109_4 |  | 83 | 0.08 | 18 | 0.11 |
| 8963109_45 |  | 87 | 0.18 | 16 | 0.28 |
| 8983948_14 |  | 30 | 0.07 | 5 | 0.2 |
| 8983948_15 |  | 117 | 0.08 | 4 | 0.5 |
| 8983948_18 |  | 328 | 0.14 | 21 | 0.54 |
| 8983948_22 |  | 89 | 0.21 | 4 | 0.33 |
| 8983948_23 |  | 100 | 0.19 | 4 | 0.32 |
| 8983948_26 |  | 169 | 0.06 | 20 | 0.2 |
| 8983948_27 |  | 160 | 0.26 | 22 | 0.3 |
| 8983948_37 |  | 56 | 0.14 | 26 | 0.34 |
| 8983948_40 |  | 67 | 0.01 | 0 | 1 |
| 8983948_7 |  | 24 | 0.2 | 8 | 0.5 |
| 8983948_8 |  | 63 | 0.11 | 1 | 1 |
| 9144311_10 |  | 108 | 0.31 | 6 | 0.47 |
| 9144311_14 |  | 83 | 0.25 | 10 | 0.5 |
| 9144311_19 |  | 124 | 0.2 | 25 | 0.35 |
| 9144311_26 |  | 110 | 0.28 | 16 | 0.57 |
| 9144311_27 |  | 70 | 0.21 | 6 | 0.5 |
| 9144311_3 |  | 20 | 0.2 | 8 | 0.5 |
| 9144311_30 |  | 86 | 0.32 | 9 | 0.54 |
| 9144311_34 |  | 65 | 0.31 | 4 | 0.54 |
| 9144311_38 |  | 80 | 0.24 | 8 | 0.37 |
| 9144311_42 |  | 12 | 0.08 | 0 | 1 |
| 10410912_10 | Lepidorhombus whiffiagonis | 39 | 0.84 | 28 | 0.89 |
| 10410912_14 |  | 18 | 0.65 | 36 | 0.55 |
| 10410912_18 |  | 9 | 0.76 | 26 | 0.76 |
| 10410912_2 |  | 20 | 0.56 | 13 | 0.7 |
| 10410912_21 |  | 20 | 0.76 | 38 | 0.79 |
| 10410912_29 |  | 35 | 0.85 | 9 | 1 |
| 10410912_6 |  | 8 | 1 | 25 | 0.83 |
| 8962643_13 |  | 43 | 0.83 | 23 | 0.84 |
| 8962643_2 |  | 144 | 0.59 | 67 | 0.61 |
| 8962643_23 |  | 69 | 0.74 | 37 | 0.8 |
| 8962643_27 |  | 57 | 0.52 | 51 | 0.78 |
| 8962643_31 |  | 72 | 0.78 | 43 | 0.82 |
| 8962643_35 |  | 41 | 0.8 | 75 | 0.76 |
| 8962643_38 |  | 78 | 0.62 | 45 | 0.84 |
| 8962643_42 |  | 56 | 0.8 | 23 | 0.85 |
| 8962643_47 |  | 45 | 0.79 | 50 | 0.84 |
| 8962643_5 |  | 23 | 1 | 12 | 1 |
| 8962643_9 |  | 80 | 0.63 | 18 | 0.65 |
| 8963109_14 |  | 190 | 0.62 | 63 | 0.65 |
| 8963109_18 |  | 230 | 0.62 | 38 | 0.65 |
| 8963109_2 |  | 450 | 0.58 | 62 | 0.6 |
| 8963109_23 |  | 96 | 0.52 | 61 | 0.55 |
| 8963109_27 |  | 283 | 0.3 | 119 | 0.57 |
| 8963109_33 |  | 16 | 0.42 | 2 | 1 |
| 8963109_4 |  | 159 | 0.72 | 81 | 0.72 |
| 8963109_45 |  | 85 | 0.65 | 56 | 0.69 |
| 8963109_6 |  | 65 | 0.08 | 72 | 0.11 |
| 8983948_15 |  | 52 | 0.76 | 11 | 0.87 |
| 8983948_18 |  | 47 | 0.85 | 8 | 0.92 |
| 8983948_22 |  | 11 | 1 | 12 | 1 |
| 8983948_23 |  | 11 | 1 | 17 | 0.97 |
| 8983948_26 |  | 6 | 1 | 13 | 0.93 |
| 8983948_27 |  | 30 | 0.95 | 12 | 1 |
| 8983948_37 |  | 0 | 1 | 15 | 0.33 |
| 8983948_38 |  | 7 | 1 | 10 | 1 |
| 8983948_40 |  | 79 | 0.86 | 3 | 0.93 |
| 8983948_8 |  | 48 | 0.75 | 25 | 1 |
| 9144311_10 |  | 88 | 0.7 | 32 | 0.78 |
| 9144311_14 |  | 27 | 0.74 | 19 | 0.83 |
| 9144311_19 |  | 8 | 1 | 41 | 0.73 |
| 9144311_26 |  | 71 | 0.62 | 14 | 0.77 |
| 9144311_27 |  | 19 | 0.56 | 27 | 0.72 |
| 9144311_3 |  | 6 | 1 | 6 | 0.83 |
| 9144311_30 |  | 44 | 0.55 | 22 | 0.71 |
| 9144311_34 |  | 33 | 0.68 | 16 | 0.79 |
| 9144311_38 |  | 9 | 0.91 | 16 | 0.92 |
| 9144311_42 |  | 81 | 0.51 | 52 | 0.63 |
| 10410912_10 | Melanogrammus aeglefinus | 19 | 1 | 31 | 1 |
| 10410912_14 |  | 12 | 1 | 10 | 1 |
| 10410912_18 |  | 18 | 0.96 | 4 | 1 |
| 10410912_2 |  | 9 | 1 | 11 | 0.95 |
| 10410912_21 |  | 36 | 0.91 | 59 | 0.77 |
| 10410912_29 |  | 36 | 0.91 | 29 | 1 |
| 10410912_6 |  | 9 | 1 | 9 | 1 |
| 8962643_13 |  | 5 | 1 | 5 | 1 |
| 8962643_2 |  | 8 | 0.83 | 5 | 1 |
| 8962643_23 |  | 2 | 1 | 3 | 1 |
| 8962643_27 |  | 15 | 0.78 | 7 | 0.82 |
| 8962643_31 |  | 40 | 0.87 | 17 | 1 |
| 8962643_35 |  | 39 | 0.81 | 25 | 0.84 |
| 8962643_38 |  | 27 | 0.63 | 0 | 1 |
| 8962643_42 |  | 12 | 0.89 | 4 | 1 |
| 8962643_47 |  | 53 | 0.65 | 5 | 1 |
| 8962643_5 |  | 7 | 1 | 7 | 1 |
| 8962643_9 |  | 11 | 0.78 | 18 | 0.79 |
| 8963109_14 |  | 1 | 1 | 1 | 1 |
| 8963109_18 |  | 0 | 1 | 3 | 1 |
| 8963109_2 |  | 0 | 1 | 1 | 1 |
| 8963109_23 |  | 0 | 1 | 8 | 0.72 |
| 8963109_27 |  | 4 | 1 | 2 | 1 |
| 8963109_4 |  | 1 | 1 | 1 | 1 |
| 8963109_45 |  | 19 | 0.91 | 3 | 1 |
| 8983948_14 |  | 31 | 0.06 | 0 | 1 |
| 8983948_15 |  | 12 | 0.08 | 0 | 1 |
| 8983948_18 |  | 110 | 0.64 | 1 | 1 |
| 8983948_22 |  | 46 | 0.79 | 7 | 1 |
| 8983948_23 |  | 10 | 0.56 | 0 | 1 |
| 8983948_26 |  | 91 | 0.78 | 3 | 1 |
| 8983948_27 |  | 0 | 1 | 1 | 1 |
| 8983948_37 |  | 21 | 0.14 | 0 | 1 |
| 8983948_38 |  | 35 | 0.29 | 1 | 1 |
| 8983948_40 |  | 202 | 0.26 | 0 | 1 |
| 8983948_8 |  | 27 | 0.11 | 1 | 1 |
| 9144311_10 |  | 1 | 1 | 0 | 1 |
| 9144311_14 |  | 6 | 0.81 | 1 | 1 |
| 9144311_19 |  | 2 | 1 | 1 | 1 |
| 9144311_26 |  | 2 | 1 | 7 | 0.8 |
| 9144311_27 |  | 2 | 1 | 2 | 1 |
| 9144311_38 |  | 5 | 0.63 | 0 | 1 |
| 10410912_10 | Rays spp | 88 | 0.39 | 35 | 0.57 |
| 10410912_14 |  | 36 | 0.56 | 47 | 0.46 |
| 10410912_18 |  | 44 | 0.54 | 46 | 0.54 |
| 10410912_2 |  | 50 | 0.54 | 24 | 0.69 |
| 10410912_21 |  | 58 | 0.5 | 135 | 0.57 |
| 10410912_29 |  | 39 | 0.6 | 32 | 0.91 |
| 10410912_6 |  | 34 | 0.51 | 32 | 0.67 |
| 8962643_13 |  | 93 | 0.35 | 39 | 0.66 |
| 8962643_2 |  | 70 | 0.63 | 110 | 0.65 |
| 8962643_23 |  | 94 | 0.54 | 123 | 0.65 |
| 8962643_27 |  | 110 | 0.44 | 81 | 0.49 |
| 8962643_31 |  | 58 | 0.5 | 61 | 0.55 |
| 8962643_35 |  | 71 | 0.76 | 54 | 0.77 |
| 8962643_38 |  | 81 | 0.74 | 49 | 0.85 |
| 8962643_42 |  | 73 | 0.72 | 52 | 0.79 |
| 8962643_47 |  | 61 | 0.76 | 64 | 0.82 |
| 8962643_5 |  | 11 | 1 | 13 | 1 |
| 8962643_9 |  | 55 | 0.65 | 68 | 0.67 |
| 8963109_14 |  | 92 | 0.61 | 31 | 0.64 |
| 8963109_18 |  | 70 | 0.45 | 112 | 0.49 |
| 8963109_2 |  | 520 | 0.04 | 196 | 0.07 |
| 8963109_23 |  | 31 | 0.46 | 1 | 1 |
| 8963109_27 |  | 24 | 0.32 | 27 | 0.38 |
| 8963109_33 |  | 1 | 1 | 0 | 1 |
| 8963109_4 |  | 143 | 0.08 | 135 | 0.11 |
| 8963109_45 |  | 106 | 0.44 | 79 | 0.51 |
| 8963109_6 |  | 29 | 0.79 | 16 | 0.79 |
| 8983948_14 |  | 35 | 0.54 | 42 | 0.84 |
| 8983948_15 |  | 73 | 0.54 | 36 | 0.94 |
| 8983948_18 |  | 44 | 0.74 | 30 | 0.86 |
| 8983948_22 |  | 48 | 0.13 | 16 | 0.25 |
| 8983948_23 |  | 73 | 0.42 | 26 | 0.51 |
| 8983948_26 |  | 59 | 0.74 | 33 | 0.78 |
| 8983948_27 |  | 113 | 0.6 | 51 | 0.62 |
| 8983948_37 |  | 53 | 0.58 | 8 | 1 |
| 8983948_38 |  | 18 | 0.82 | 15 | 0.84 |
| 8983948_40 |  | 67 | 0.34 | 9 | 0.67 |
| 8983948_7 |  | 22 | 1 | 24 | 1 |
| 8983948_8 |  | 27 | 1 | 25 | 1 |
| 9144311_10 |  | 23 | 0.85 | 30 | 0.89 |
| 9144311_14 |  | 30 | 0.9 | 31 | 0.93 |
| 9144311_19 |  | 46 | 0.8 | 30 | 0.83 |
| 9144311_26 |  | 91 | 0.49 | 21 | 0.7 |
| 9144311_27 |  | 82 | 0.37 | 40 | 0.6 |
| 9144311_3 |  | 81 | 0.54 | 28 | 0.71 |
| 9144311_30 |  | 139 | 0.41 | 32 | 0.62 |
| 9144311_34 |  | 75 | 0.48 | 40 | 0.65 |
| 9144311_38 |  | 86 | 0.39 | 65 | 0.48 |
| 9144311_42 |  | 51 | 0.54 | 13 | 0.67 |
