# Supplementary material for: Toward elimination of unwanted catches using a 100 mm T90 extension and codend in demersal mixed fisheries
Source: PLoS One. 2020 Jul 8;15(7):e0235368. doi: 10.1371/journal.pone.0235368 (PMC7343172; doi:10.1371/journal.pone.0235368)
Supplement: S3 Table — (DOCX) [file pone.0235368.s003.docx]

S3 Table. Mean observed weight (in kg) per species in the wanted catches (LAN) and unwanted catches (DIS)

|  |  | Control trawl | | T90 Experimental trawl | |
| --- | --- | --- | --- | --- | --- |
| Species | Catch category | Mean Weight | Sd | Mean Weight | Sd |
| Argentina silus | DIS | 0.88 | 0.99 | 1.09 | 1.02 |
| Cancer pagurus | DIS | 11.99 | 8.67 | 8.25 | 6.72 |
| Capros aper | DIS | 13.62 | 20.2 | 2.32 | 3.88 |
| Gadus morhua | LAN | 7.5 | 8.24 | 5.33 | 3.54 |
| Gurnards spp | DIS | 13.99 | 8.72 | 4.06 | 4.85 |
| Gurnards spp | LAN | 2.87 | 2.28 | 2.9 | 1.85 |
| Illex coindetii | DIS | 5.05 | 6.13 | 1.75 | 1.86 |
| Lepidorhombus whiffiagonis | DIS | 8.5 | 11.54 | 3.97 | 3.64 |
| Lepidorhombus whiffiagonis | LAN | 6.59 | 4.04 | 5.7 | 3.85 |
| Loligo spp | DIS | 1.57 | 1.86 | 0.51 | 0.26 |
| Loligo spp | LAN | 4.18 | 5.88 | 2.81 | 3.81 |
| Lophius | DIS | 18.42 | 13.19 | 14.84 | 12.29 |
| Lophius | LAN | 105.8 | 42.89 | 103.68 | 38.51 |
| Melanogrammus aeglefinus | DIS | 10.39 | 14.16 | 1.83 | 1.01 |
| Melanogrammus aeglefinus | LAN | 7.41 | 6.22 | 7.66 | 9.82 |
| Merluccius merluccius | LAN | 5.1 | 4.5 | 4.68 | 3.79 |
| Microstomus kitt | LAN | 1.3 | 1.47 | 1.47 | 1.81 |
| Molva molva | LAN | 11.7 | 3.86 | 8.39 | 8.88 |
| Mullus surmuletus | LAN | 0.67 | 0.42 | 0.77 | 0.66 |
| Rays spp | DIS | 12.11 | 9.92 | 7.41 | 5.81 |
| Rays spp | LAN | 20.65 | 17.41 | 19.35 | 17.13 |
| Scyliorhinus canicula | DIS | 7.06 | 7.04 | 3.47 | 2.71 |
| Trachurus trachurus | DIS | 2.52 | 2.34 | 1.01 | 0.5 |
| Trisopterus spp | DIS | 2.12 | 1.69 | 0.14 | 0.13 |
| Zeus faber | LAN | 4.15 | 7.28 | 3.25 | 3.4 |
